# Supplementary material for: Characterization of Critical Determinants of ACE2–SARS CoV-2 RBD Interaction
Source: Int J Mol Sci. 2021 Feb 25;22(5):2268. doi: 10.3390/ijms22052268 (PMC7956771; doi:10.3390/ijms22052268)
Supplement: Supplementary file 1 [file ijms-22-02268-s001.pdf]

Supplemental Table 1

Insert	DNA sequence	Amino Acid sequence
SmBiT-ACE2 (IL12 secretion signal-ACE2-HA-His)	ATGTGCCCCCAAAAGTTGACCAT<br>CTCATGGTTCGCAATTGTACTACT<br>AGTGAGTCCCTTGATGGCAGGCG<br>GAAGTGGAGGTTCCTCTGGAGTG<br>ACCGGATACAGGCTGTTCGAGGA<br>GATCCTGCAGAGCACAATCGAG<br>GAGCAGGCCAAGACCTTCCTGG<br>ACAAGTTTAACCACGAGGCCGA<br>GGATCTGTTTTATCAGAGCTCCCT<br>GGCCTCCTGGAACTACAATACCA<br>ACATCACAGAGGAGAATGTGCA<br>GAACATGAACAATGCCGGCGAC<br>AAGTGGTCTGCCTTCCTGAAGGA<br>GCAGAGCACACTGGCCCAGATG<br>TACCCTCTGCAGGAGATCCAGAA<br>CCTGACCGTGAAGCTGCAGCTGC<br>AGGCCCTGCAGCAGAATGGCTCT<br>AGCGTGCTGTCCGAGGATAAGTC<br>TAAGCGGCTGAATACAATCCTGA<br>ACACCATGAGCACAATCTATTCC<br>ACCGGCAAGGTGTGCAATCCCG<br>ACAACCCTCAGGAGTGTCTGCTG<br>CTGGAGCCTGGCCTGAATGAGAT<br>CATGGCCAACAGCCTGGATTACA	MCPQKLTISW<br>FAIVLLVSPLM<br>AGGSGGSSGV<br>TGYRLFEEILQS<br>TIEEQAKTFLD<br>KFNHEAEDLF<br>YQSSLASWNY<br>NTNITEENVQ<br>NMNNAGDK<br>WSAFLKEQSTL<br>AQMYPLQEIQ<br>NLTVKLQLQA<br>LQQNGSSVLSE<br>DKSKRLNTILN<br>TMSTIYSTGKV<br>CNPDNPQECL<br>LLEPGLNEIMA<br>NSLDYNERLW<br>AWESWRSEVG<br>KQLRPLYEEYV<br>VLKNEMARA<br>NHYEDYGDY<br>WRGDYEVNG<br>VDGYDYSRGQ

	ATGAGAGGCTGTGGGCATGGGA	LIEDVEHTFEEI
	GTCCTGGCGCTCTGAAGTGGGCA	KPLYEHLHAY
	AGCAGCTGCGGCCACTGTACGA	VRAKLMNAYP
	GGAGTATGTGGTGCTGAAGAACG	SYISPIGCLPAH
	AGATGGCCAGAGCCAATCACTA	LLGDMWGRF
	CGAGGACTATGGCGATTACTGGA	WTNLYSLTVPF
	GGGGCGACTATGAGGTGAACGG	GQKPNIDVTD
	CGTGGACGGCTACGATTATAGCC	AMVDQAWDA
	GCGGCCAGCTGATCGAGGATGTG	QRIFKEAEKFF
	GAGCACACATTCGAGGAGATCA	VSVGLPNMTQ
	AGCCCCTGTATGAGCACCTGCAC	GFWENSMLTD
	GCCTACGTGCGGGCCAAGCTGAT	PGNVQKAVC
	GAATGCCTATCCAAGCTACATCT	HPTAWDLGK
	CCCCAATCGGATGCCTGCCTGCA	GDFRILMCTK
	CACCTGCTGGGCGACATGTGGGG	VTMDDFLTAH
	CAGATTCTGGACAAACCTGTACT	HEMGHIQYD
	CCCTGACCGTGCCATTTGGCCAG	MAYAAQPFLL
	AAGCCCAATATCGACGTGACCG	RNGANEGFHE
	ATGCCATGGTGGACCAGGCCTGG	AVGEIMSLSAA
	GATGCCCAGAGGATCTTCAAGGA	TPKHLKSIGLL
	GGCCGAGAAGTTCTTCGTGAGCG	SPDFQEDNETE
	TGGGCCTGCCAAACATGACACA	INFLLKQALTI
	GGGCTTTTGGGAGAATAGCATGC	VGTLPFTYMLE
	TGACCGACCCAGGCAACGTGCA	KWRWMVFKG
	GAAGGCCGTGTGCCACCCAACA	EIPKDQWMKK
	GCATGGGACCTGGGCAAGGGCG	WWEMKREIVG
	ATTTCCGCATCCTGATGTGCACC	VVEPVPHDET
	AAGGTGACAATGGACGATTTTCT	YCDPASLFHVS
	GACCGCCCACCACGAGATGGGC	NDYSFIRYYTR

	CACATCCAGTATGATATGGCATA<br>CGCAGCACAGCCCTTCCTGCTGA<br>GGAATGGCGCCAACGAGGGCTTT<br>CACGAGGCCGTGGGCGAGATCA<br>TGTCTCTGAGCGCCGCCACACCT<br>AAGCACCTGAAGAGCATCGGCC<br>TGCTGTCCCCAGACTTCCAGGAG<br>GATAACGAGACCGAGATCAATTT<br>TCTGCTGAAGCAGGCCCTGACCA<br>TCGTGGGCACACTGCCCTTCACC<br>TATATGCTGGAGAAGTGGCGCTG<br>GATGGTGTTTAAGGGCGAGATCC<br>CTAAGGACCAGTGGATGAAGAA<br>GTGGTGGGAGATGAAGCGGGAG<br>ATCGTGGGCGTGGTGGAGCCTGT<br>GCCACACGACGAGACCTACTGTG<br>ATCCTGCCAGCCTGTTCCACGTG<br>TCTAACGACTATAGCTTTATCAG<br>GTACTATACCCGCACACTGTACC<br>AGTTCCAGTTTCAGGAGGCCCTG<br>TGCCAGGCAGCAAAGCACGAGG<br>GCCCACTGCACAAGTGTGATATC<br>TCCAACTCTACAGAGGCCGGCCA<br>GAAGCTGTTCAATATGCTGAGAC<br>TGGGCAAGTCCGAGCCATGGAC<br>CCTGGCCCTGGAGAACGTGGTGG<br>GAGCCAAGAATATGAACGTGAG<br>GCCTCTGCTGAATTATTTCGAGC<br>CACTGTTTACATGGCTGAAGGAC	TLYQFQFQEAL<br>CQAAKHEGPL<br>HKCDISNSTEA<br>GQKLFNMLRL<br>GKSEPWTLAL<br>ENVVGAKNM<br>NVRPLLNYFEP<br>LFTWLKDQNK<br>NSFVGWSTDW<br>SPYADQSIKVR<br>ISLKSALGDKA<br>YEWNDNEMY<br>LFRSSVAYAM<br>RQYFLKVKNQ<br>MILFGEEDVRV<br>ANLKPRISFNF<br>FVTAPKNVSDI<br>IPRTEVEKAIR<br>MSRSRINDAFR<br>LNDNSLEFLGI<br>QPTLGPPNQP<br>PVSGSGYPYDV<br>PDYAYPYDVP<br>DYAYPYDVPD<br>YASSGHIEGRH<br>MLEMGHHHH<br>HHHHHH

	CAGAATAAGAACTCCTTTGTGGG<br>CTGGTCCACCGACTGGTCTCCAT<br>ACGCCGATCAGTCTATCAAGGTG<br>AGGATCTCTCTGAAGAGCGCCCT<br>GGGCGACAAGGCCTATGAGTGG<br>AATGATAACGAGATGTACCTGTT<br>CAGATCCTCTGTGGCCTATGCCA<br>TGAGGCAGTACTTCCTGAAGGTG<br>AAGAACCAGATGATCCTGTTTGG<br>CGAGGAGGACGTGCGGGTGGCC<br>AATCTGAAGCCCAGAATCAGCTT<br>TAACTTCTTTGTGACAGCCCCCA<br>AGAACGTGAGCGATATCATCCCT<br>CGCACCGAGGTGGAGAAGGCCA<br>TCCGGATGAGCCGGTCCAGAATC<br>AACGACGCCTTCAGACTGAATGA<br>TAACAGCCTGGAGTTTCTGGGCA<br>TCCAGCCTACCCTGGGACCACCT<br>AATCAGCCACCCGTGTCTGGCAG<br>CGGCTACCCATATGACGTGCCCG<br>ATTACGCCTATCCTTACGACGTG<br>CCAGATTATGCCTACCCCTATGA<br>CGTGCCTGATTACGCAAGCTCCG<br>GACACATCGAGGGCAGGCACAT<br>GCTGGAGATGGGCCATCATCACC<br>ATCATCACCACCACCACCACTGA	
RBD-LgBiT	ATGGAGACAGACACACTCCTGCTA<br>TGGGTACTGCTGCTCTGGGTTCCAG	METDTLLL<br>WVLLLWVP

(IgK leader-3XFLAG-RBD-LgBiT-His)	GTTCCACTGGTGACTCTGGCTCTAG	GSTGDSGSS
	CGGCTCTGGCAGCGGCGACTACAA	GSGSGDYK
	GGACCACGACGGTGACTACAAGGA	DHDGDYKD
	CCACGACATCGACTACAAGGACGA	HDIDYKDD
	CGACGACAAGGGAGGAGGAGGCT	DDKGGGGS
	CTAGCGGCGGCAACATCACAAATC	SGGNITNLC
	TGTGCCCATTCGGCGAGGTGTTTAA	PFGEVFNAT
	CGCCACCAGATTTGCCAGCGTGTAT	RFASVYAW
	GCCTGGAACCGGAAGAGAATCTCT	NRKRISNCV
	AATTGCGTGGCCGACTATAGCGTGC	ADYSVLYNS
	TGTACAATAGCGCCTCCTTCTCTAC	ASFSTFKCY
	CTTTAAGTGCTATGGCGTGTCCCCC	GVSPTKLND
	ACAAAGCTGAACGACCTGTGCTTC	LCFTNVYA
	ACCAACGTGTACGCCGACTCTTTTG	DSFVIRGDE
	TGATCAGGGGCGATGAGGTGCGCC	VRQIAPGQT
	AGATCGCACCTGGACAGACAGGCA	GKIADYNY
	AGATCGCCGACTACAACTATAAGC	KLPDDFTGC
	TGCCAGACGATTTCACCGGCTGCGT	VIAWNSNN
	GATCGCCTGGAATAGCAACAATCT	LDSKVGGN
	GGATTCCAAAGTGGGCGGCAACTA	YNYLYRLFR
	CAATTATCTGTACCGGCTGTTCAGA	KSNLKPFER
	AAGAGCAACCTGAAGCCCTTTGAG	DISTEIYQAG
	CGGGATATCAGCACAGAGATCTAC	STPCNGVEG
	CAGGCAGGCTCCACCCCTTGCAAC	FNCYFPLQS
	GGAGTGGAGGGCTTCAATTGTTATT	YGFQPTNG
	TTCCCCTGCAGAGCTACGGCTTCCA	VGYQPYRV
	GCCTACAAATGGCGTGGGCTATCA	VVLSFELLH
	GCCATACAGGGTGGTGGTGCTGTCC	APATVGSSG
	TTTGAGCTGCTGCACGCACCTGCAA	GGGSGGGG

	CCGTGGGGAGTTCCGGTGGTGGCG<br>GGAGCGGAGGTGGAGGCTCGAGCG<br>GTGGAGTGTTCACACTGGAGGACTT<br>TGTGGGCGATTGGGAGCAGACCGC<br>CGCCTACAACCTGGACCAGGTGCT<br>GGAGCAGGGAGGCGTGTCCTCTCT<br>GCTGCAGAATCTGGCCGTGTCTGTG<br>ACACCAATCCAGAGGATCGTGCGC<br>AGCGGCGAGAACGCCCTGAAGATC<br>GACATCCACGTGATCATCCCCTACG<br>AGGGCCTGTCCGCCGATCAGATGG<br>CCCAGATCGAGGAGGTGTTCAAGG<br>TGGTGTATCCAGTGGACGATCACCA<br>CTTCAAAGTGATCCTGCCCTACGGC<br>ACCCTGGTCATCGACGGAGTGACC<br>CCCAACATGCTGAATTATTTCGGCC<br>GGCCTTACGAGGGCATCGCCGTGTT<br>TGATGGCAAGAAGATCACCGTGAC<br>AGGCACCCTGTGGAACGGCAATAA<br>GATCATCGACGAGCGGCTGATCAC<br>ACCTGATGGCTCCATGCTGTTCAGA<br>GTGACCATCAACTCCTCCTCTGGAC<br>ACATCGAGGGCCGCCACATGCTGG<br>AGATGGGCCATCATCACCATCATC<br>ACCACCACCACCACTGA	SSGGVFTLE<br>DFVGDWEQ<br>TAAYNLDQ<br>VLEQGGVSS<br>LLQNLAVSV<br>TPIQRIVRSG<br>ENALKIDIH<br>VIIPYEGLSA<br>DQMAQIEE<br>VFKVVYPVD<br>DHHFKVILP<br>YGTLVIDGV<br>TPNMLNYF<br>GRPYEGIAV<br>FDGKKITVT<br>GTLWNGNK<br>IIDERLITPD<br>GSMLFRVTI<br>NSSSGHIEG<br>RHMLEMGH<br>HHHHHHH<br>HH
